# Adult Vaccine Hesitancy Scale in Arabic and French: Protocol for Translation and Validation in the World Health Organization Eastern Mediterranean Region

**DOI:** 10.2196/36928

**Published:** 2022-04-12

**Authors:** Radwa Nour, Leigh Powell, Wafa K Alnakhi, Heba Mamdouh, Youness Zidoun, Hamid Y Hussain, Hanan Al Suwaidi, Nabil Zary

**Affiliations:** 1 Institute for Excellence in Health Professions Education Mohammed Bin Rashid University of Medicine and Health Sciences Dubai United Arab Emirates; 2 Department of Data Analysis, Research and Studies Dubai Health Authority Dubai United Arab Emirates; 3 College of Medicine Mohammed Bin Rashid University of Medicine and Health Sciences Dubai United Arab Emirates

**Keywords:** scale, instrument, vaccine hesitancy, COVID-19, validation, translation, Arabic, French, EMRO

## Abstract

**Background:**

The world as we know it changed during the COVID-19 pandemic. Hope has emerged with the development of new vaccines against the disease. However, many factors hinder vaccine uptake and lead to vaccine hesitancy. Understanding the factors affecting vaccine hesitancy and how to assess its prevalence have become imperative amid the COVID-19 pandemic. The vaccine hesitancy scale (VHS), developed by the World Health Organization (WHO) Strategic Advisory Group of Experts on Immunization, has been modified to the adult VHS (aVHS) and validated in English and Chinese. To our knowledge, no available aVHS has been designed or validated in Arabic or French.

**Objective:**

The aim of this research is to translate the aVHS from its original English language to Arabic and French and validate the translations in the WHO Eastern Mediterranean region.

**Methods:**

The study will follow a cross-sectional design divided into 5 phases. In phase 1, the original aVHS will be forward-translated to Arabic and French, followed by backward translation to English. An expert committee will review and rate all versions of the translations. Expert agreement will then be measured using the Cohen kappa coefficient (k). In phase 2, the translated aVHS will be pilot-tested with 2 samples of participants (n=100): a group that speaks both Arabic and English and another that speaks French and English. Participants’ responses to the English version will also be collected. In phase 3, responses will then be compared. Descriptive statistics and paired t tests or one-way analyses of variance (ANOVA) and Pearson correlation coefficient will be used in the preliminary validation. In phase 4, prefinal versions (Arabic and French) will be tested with larger sample sizes of Arabic speakers (n=1000) and French speakers (n=1000). Sociodemographic information and vaccination status will be collected and used for further analysis. In phase 5, the scale's statistical reliability and internal consistency will be measured using Cronbach alpha. An exploratory factor analysis (EFA) and confirmatory factor analysis (CFA) will be used to examine the model fit resulting from the EFA. ANOVA and regression models will be constructed to control for confounders. All data will be electronically collected.

**Results:**

As of January 2022, the scale had been translated to Arabic and French and was undergoing the process of back translation. All data collection tools have been prepared (ie, sociodemographics, vaccination status, and open-ended questions) and are ready to go into their electronic formats. We expect to reach the desired sample size in this phase by June 2022.

**Conclusions:**

This study will provide researchers with a validated tool to assess adult vaccine hesitancy within populations that speak Arabic and/or French and provide a road map to scale translation and ensure cross-cultural adaptation.

**International Registered Report Identifier (IRRID):**

PRR1-10.2196/36928

## Introduction

### Background

The world as we know it changed during the COVID-19 pandemic. Although hope has emerged with the development of new vaccines against the disease, public health providers still face a significant challenge in ensuring uptake of those vaccines by the larger public. To achieve herd immunity to COVID-19, a substantial proportion of the population would need to be vaccinated [1]. However, there are many factors hindering vaccine uptake, including logistic and economic factors and misinformation leading to a lack of public confidence in the effectiveness and safety of the vaccines [1,2]. These are all factors that contribute to what has been termed vaccine hesitancy. Vaccine hesitancy is defined as the “refusal or delay of the uptake of vaccines despite the availability of services” [3,4]. Vaccine hesitancy is a global problem that has negatively impacted public health. The World Health Organization (WHO) Strategic Advisory Group of Experts on Immunization (SAGE) established a working group to address this issue [3].

Understanding the factors affecting vaccine hesitancy and how to assess its prevalence have become imperative amid the COVID-19 pandemic. Developing new vaccines utilizing existing or new technologies spurred public scrutiny regarding the need, safety, and efficacy of such vaccines [1,2]. This prompted a need for a tool to help assess vaccine hesitancy among adults. Before this, vaccine hesitancy scales (VHS) available in the literature focused on parental attitudes and perceptions regarding vaccinating their children [5]. Various attempts were made to develop or adapt parental VHS tools for adults, but very few went through the rigor of being validated [5-8]. The need for such a validated scale has only increased with the COVID-19 pandemic.

Various scales have been developed to measure hesitancy among parents or health care workers [5]. The WHO SAGE Working Group on Vaccine Hesitancy developed a 10-item VHS that is widely used in different countries and settings [9]. The VHS has been modified to the adult VHS (aVHS) and has been adapted and validated in English and Chinese [5].

The aVHS is a 10-item scale with a 5-point Likert scale ranging from “Strongly disagree” to “Strongly agree.” The Likert scale items have scores ranging from 10 to 50, where 50 represents the highest degree of vaccine hesitancy and 10 represents the lowest. The scores on 7 items on the scale will be reverse coded so that the highest scores reflect the highest degree of vaccine hesitancy. A cut-off score of 24 is used to dichotomize the outcome into “vaccine hesitant” and “not vaccine hesitant” categories. The score range and cut-off score have been proposed and validated by the research team developing the scale [5].

### Objective

To our knowledge, no available aVHS has been designed or validated in Arabic or French.

Both of these languages are widely used in the countries included in the WHO Regional Office of the Eastern Mediterranean (EMRO) [10], with Arabic being the most commonly used language in EMRO and French being a commonly used language in EMRO countries such as Algeria, Tunisia, Morocco, and Djibouti. For the aVHS to be valid for these populations, the scale must undergo translation and cultural adaptation prior to validation and reliability testing. This study will adopt a rigorous methodology to translate and validate the aVHS and conduct a proper psychometric evaluation of the translated version to make the scale available to all scholars in the EMRO region and beyond. In addition, we plan to survey a representative sample of the region to assess the aVHS’ structure, internal consistency, and validity across different social and demographic settings. Therefore, this research aims to translate the aVHS from its original English language to Arabic and French and validate the translations in the WHO Eastern Mediterranean region.

## Methods

**Figure 1:**
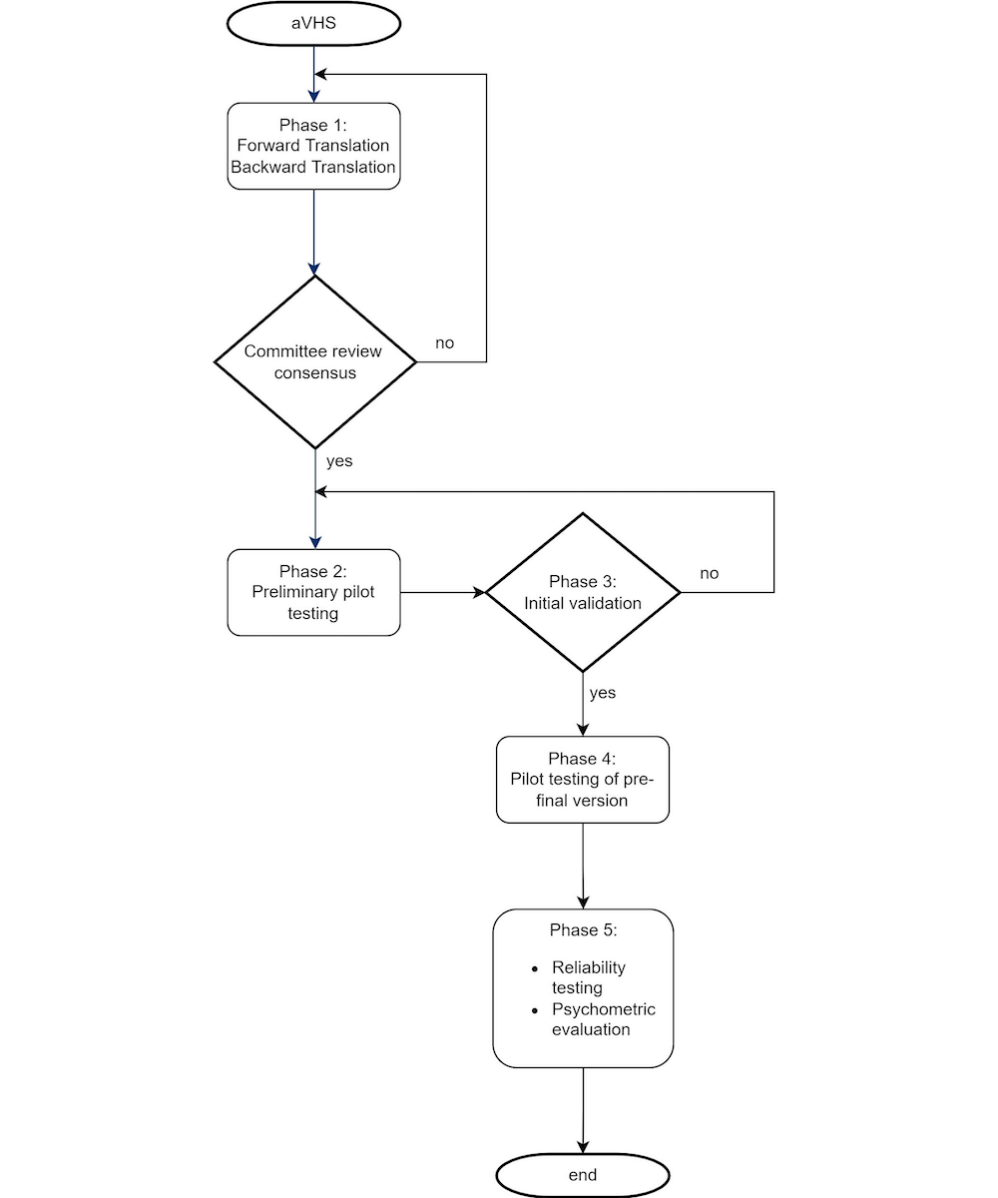
Flow diagram of study phases. aVHS: adult vaccine hesitancy scale.

The study will follow a cross-sectional design divided into 5 phases inspired by the methodology proposed in the “Guidelines for developing, translating, and validating a questionnaire in perioperative and pain medicine” [11]. Study phases are detailed in the following sections and illustrated in Figure 1.

### Study Phases

#### Phase 1: Translation

The original aVHS will be forward translated to Arabic by individuals who are bilingual in Arabic and English and translated to French by individuals who are bilingual in French and English. Translators’ mother tongues are the language of translation in order to capture the target language's nuances better. Backward translation will be conducted to reveal any misunderstanding or miswording in the forward translation. A different set of bilingual individuals (Arabic and English; French and English) will conduct the back translation into their mother tongues. To avoid bias, back‑translators will not be aware of the intended purpose of the scale and will be blinded to the English version. A review of all versions of the translations and any discrepancies will be reviewed and resolved by an expert review committee of 6 to 10 members [12]. The committee will be required to rate each item for relevance, representativeness, and technical quality on a scale of (accept—reject—modify). Expert agreement will then be measured using the Cohen kappa coefficient; a cut-off of ≥0.70 will be deemed acceptable [13]. The translation and back-translation process will be repeated until a satisfactory agreement is reached.

The data collection tools for this study will include both the English and translated versions of the aVHS (Table 1).

**Table 1:** Data collection tools for each testing phase.

Data collection tool	Preliminary pilot testing phase	Testing of prefinal version
aVHS^a^ (Translated)	x^b^	x
aVHS (English)	x	—^c^
A set of open‑ended questions	x	—
Sociodemographics	—	x
Vaccination status	—	x

^a^aVHS: adult vaccine hesitancy scale.

^b^Tool will be used.

^c^Tool will not be used.

#### Phase 2: Preliminary Pilot Testing

The translated aVHS will be pilot tested with 2 samples of participants: a group that speaks both Arabic and English and another that speaks French and English. Based on previous studies, at least 5 participants per scale item are required for testing [5,14]. As such, our sample size will be 100, equally divided between the Arabic and French translations. A convenience sampling approach will be utilized. Participants will be approached on the Mohammed Bin Rashid University (MBRU) campus and screened for inclusion criteria. They will be asked to complete the translated scale and subsequently requested (verbally by an interviewer) to elaborate on what they thought each scale item meant. This process helps ensure that the translated items retain the same meaning as intended in the English scale and ensure there is no confusion regarding the translated scale. After participants complete this process using the translated aVHS, participants will be asked to complete the aVHS in English. Items on the original English scale will be in a different order from that of the translated version [12]. Therefore, participants will be asked to complete the translated version first without seeing the original scale.

#### Phase 3: Initial Validation Phase

Responses to both the English and translated versions of the scale will then be compared. Statistical analyses will be conducted to test the reliability of the translation against the original scale. Descriptive statistics and paired *t* tests or 1-way analyses of variance (ANOVA) will be used to analyze the data collected. The Pearson correlation coefficient will be used in preliminary validation of translated versions against the original version of the scale. If reliability is not achieved, then phase 2 will be repeated. If the translated versions are statistically reliable, these will become the prefinal versions for phase 4.

#### Phase 4: Testing of the Prefinal Version

Prefinal versions (Arabic and French) will be tested with a larger sample. Generally, there is a lack of consensus on the sample size required for validity testing of a scale [15]. A review of the research in this area found it has been recommended to use at least 10 participants per item when doing a comprehensive psychometric analysis [12,16] and that between 300 and 500 participants are required to perform an exploratory factor analysis (EFA) [12], which will be conducted in this study. A sample size of 1000 participants was deemed “excellent” for scale validation [12,17]; therefore, our proposed sample size is 1000 Arabic speakers and 1000 French speakers. This sample size is set to avoid sampling errors that may reduce the statistical power needed to validate the scale [18]. Data about participants, such as sociodemographic information and vaccination status, will also be collected (as detailed in Table 2) and used for further analysis.

Data will be collected anonymously via a link to an online survey hosted locally at MBRU. The link will be posted on different social media platforms. A series of questions to determine participant eligibility will be asked. Those who meet the inclusion criteria will be able to proceed to data collection.

**Table 2:** Variable names and types.

Variable name	Measurement level	Description/parameter
Age (years)	Categorical	18-25, 26-35, 36-45, 46-55, 56-65, 66-75, ≥76
Gender	Categorical	Male, female, other
Occupation [19]	Categorical	1. Managers; 2. Professionals; 3. Technicians and associate professionals; 4. Clerical support workers; 5. Services and sales workers; 6. Skilled agriculture, forestry, and fishery workers; 7. Craft and related trade workers; 8. Plant and machine operators and assemblers; 9. Elementary occupations; 0. Armed forces occupations
Nationality	Categorical	Based on the World Bank list of countries [20]
Income (US $)	Numerical	Annual
aVHS^a^	5-point Likert scale	Strongly disagree to strongly agree

^a^aVHS: adult vaccine hesitancy scale.

#### Phase 5: Validation of the Prefinal Version (Psychometric Testing)

The scale’s statistical reliability and internal consistency will be measured using Cronbach alpha; values range from 0 to 1, where a value of 0 indicates no internal consistency and a value of 1 reflects perfect internal consistency. A Cronbach alpha cut-off of 0.70 will indicate adequate internal consistency [21]. An EFA will be conducted to examine the structure of the scale as well as reliability of the items. Confirmatory factor analysis (CFA) will be used to examine model fit resulting from the EFA. ANOVA or regression models will be constructed to evaluate potential confounding effects arising from variations in sociodemographic factors of the participants. Continuous variables will be further categorized after data visualization to avoid groups with sparse data. Mean, medians, IQRs, and SDs will be used to describe continuous variables. For categorical or non-normally distributed variables, the Wilcoxon Signed Rank test will be used. Frequency distributions, percentages, and chi-square tests will describe binary and categorical variables and identify significant differences between groups. The correlation between vaccine hesitancy scores and the vaccination status reported by participants will also be explored. Analyses will be conducted and reported for both Arabic and French versions independently.

All data will be electronically collected (ie, templates, devices) to ensure the standardization of the data collection process and minimize errors from manual data entry.

### Privacy, Confidentiality, and Data Storage

No identifying information will be collected from participants in any of the study phases. Data will be collected and stored on local MBRU servers. Data collected for the validation of the prefinal version phase will be anonymously collected via a link to an online survey hosted on a website hosted locally. The link will be posted on different social media platforms, and only the research team will have access to the data.

The study is part of the research project titled “Addressing Vaccine Hesitancy through Targeted and Personalized Mobile Educational Interventions for Different Populations in the Eastern Mediterranean region.”

### Ethical Approval

The study has been reviewed and approved by the institutional review board of Mohammed Bin Rashid University of Medicine and Health Sciences (MBRU IRB-2021-87).

### Data Analysis

Data analysis in each phase will follow the steps explained in the preceding paragraphs. Statistical analysis software will be used to analyse the data set. A complete case analysis approach will be adopted. Data will be tested for normality, visually using histograms and statistically using the Shapiro-Wilk test. The significance level cut-off will be set at *P*<.05*,* and exact *P* values will be reported.

## Results

As of January 2022, the scale had been translated to Arabic (Multimedia Appendix 1) and French (Multimedia Appendix 2) and was undergoing back translation. The original English version can also be found in Multimedia Appendix 3. All data collection tools have been prepared (ie, sociodemographics, vaccination status, and open-ended questions) and are ready to go into their electronic formats.

This project includes 3 stages of data collection and analysis: (1) expert agreement on translation; (2) initial validation phase where responses on both versions of the scale will be compared; and (3) psychometric testing of the prefinal version in which internal consistency, validity, and model fit will be explored and tested. We expect to reach the desired sample size in this phase (2000 participants) by June 2022.

All translated versions of the scale will be publicly available to scholars free of charge.

## Discussion

### Overview

This study is part of a group of studies conducted by the Institute for Excellence in Health Professions Education (ieHPE) at MBRU, located in Dubai, United Arab Emirates. These studies are all taking different approaches to address vaccine hesitancy. The original, English-language version of the aVHS is being used as part of these studies (unpublished). We selected the aVHS as it is one of very few tools to have undergone validation prior to implementation. In this project, we will be translating and validating the scale for countries included in the WHO EMRO [10], in which most of the population speak Arabic and/or French. We reviewed the literature and found existing VHS in Arabic; however, none of them have undergone a rigorous validation process [22-25]. Such a process is needed to clarify the underpinnings of the scale factor loadings, structure, and stability. For example, suppose we aim to understand vaccine hesitancy in our region. In that case, we cannot simply rely upon scales that have been validated in a different setting; surveys, questionnaires, and scales, in general, cannot be validated unless they have been validated and tested on the population in which they are planned to be used [26]. Our objective is to adopt a rigorous methodology to ensure validity and reliability of the tool.

Sample size assessment was also an issue we encountered while planning the study. There is no clear consensus in the literature for the sample size calculation required for scale validation. For example, published studies mentioned that 1000 was an ideal sample size [12,17], while others reported that 10 subjects per item would be sufficient [11]. This would have reduced the proposed sample size from 1000 to 100 for this 10-item scale study. It was also previously mentioned that 300 to 500 subjects are required to perform an EFA [12]. We chose the sample size for this study because it ensures we will be able to conduct the analysis proposed.

Our literature review also uncovered that sociodemographic factors and differences in the political climate are potentially reflected in vaccine hesitancy status [3,26,27]. Interestingly, although overall health in general has commonly been associated with higher educational level [3], those with higher educational levels have been shown to have a greater hesitancy to vaccinate [3], even among health care providers [27], demonstrating that underlying social determinants are different than with overall health. The sociodemographic factors that are correlated with vaccine hesitancy have been explored in some studies in the region [28]. Still, most have focused on a single country [25], while other studies did not address these factors [24,29]. This study aims to explore those factors across different countries in the region. In addition, the relationship between vaccine hesitancy and actual vaccination will be explored and reported in this study. Although some countries mandate vaccination for their citizens and some pose some restrictions on unvaccinated individuals, vaccine hesitancy as a perspective might be independent of actual vaccination. This is a meaningful relationship to explore to understand the population’s attitude toward vaccines and actual behaviors potentially influenced by governments’ mandates.

### Limitations

One of the limitations of the protocol is the convenience sampling approach utilized. Although convenience sampling could potentially introduce bias to the study results, it is still a practical approach utilized by various studies, especially in the absence of a sampling frame. We expect this to be a minor issue in phase 3 of the study as this phase aims to test the translation only. As for phase 4, data stratification and regression models will allow us to elucidate and control for potential bias. Another limitation is posting the survey online. We are aware that this impacts the generalizability of the results, since only people with electronic devices, internet connection, and a degree of digital literacy will be able to access the survey and may not be representative of the rest of the population. But since this protocol proposes translating and validating the scale and does not aim at measuring the prevalence of vaccine hesitancy, we hypothesize that the impact should be minor.

### Conclusions

This study will provide researchers with a validated tool to assess adult vaccine hesitancy within populations that speak Arabic or French and provide a road map on how to scale translation and ensure cross-cultural adaptation. We aim at supporting our approach with statistical evidence.

Any work resulting from this project will be disseminated nationally and internationally through submission to academic journals and international conferences.

## Appendix

Multimedia Appendix 1 Arabic translation of the adult vaccine hesitancy scale (aVHS).

Multimedia Appendix 2 French translation of the adult vaccine hesitancy scale (aVHS).

Multimedia Appendix 3 Original English version of the adult vaccine hesitancy scale (aVHS).

## Abbreviations

ANOVA: analysis of variance
aVHS: adult vaccine hesitancy scale
CFA: confirmatory factor analysis
EFA: exploratory factor analysis
EMRO: Regional Office for the Eastern Mediterranean
ieHPE: Institute for Excellence in Health Professions Education
MBRU: Mohammed Bin Rashid University
SAGE: Strategic Advisory Group of Experts on Immunization
VHS: vaccine hesitancy scale
WHO: World Health Organization
