# Supplementary material for: Adult Vaccine Hesitancy Scale in Arabic and French: Protocol for Translation and Validation in the World Health Organization Eastern Mediterranean Region
Source: JMIR Res Protoc. 2022 Apr 12;11(4):e36928. doi: 10.2196/36928 (PMC9007230; doi:10.2196/36928)
Supplement: Multimedia Appendix 1 [file resprot_v11i4e36928_app1.docx]

**Appendix I**

**Arabic translation of aVHS**

**Unvalidated version**

| موافق بشدة | موافق | محايد | غير موافق | غير موافق بشدة | العبارات |
| --- | --- | --- | --- | --- | --- |
|  |  |  |  |  | التطعيم مهم Arلصحتي |
|  |  |  |  |  | التطعيمات فعالة |
|  |  |  |  |  | كوني مطعماً مهم لصحة الآخرين في مجتمعي |
|  |  |  |  |  | كل التطعيمات التي توصي بها الجهات الصحية الرسمية بالدولة مفيدة |
|  |  |  |  |  | التطعيمات الجديدة تحمل مخاطر أكثر من التطعيمات القديمة |
|  |  |  |  |  | المعلومات التي أتلقاها حول التطعيمات من الجهات الصحية الرسمية بالدولة جديرة بالثقة (موثوق بها) |
|  |  |  |  |  | الحصول على التطعيمات طريقة جيدة لحمايتي من الأمراض |
|  |  |  |  |  | بشكل عام، أفعل ما يوصي به طبيبي أو مقدم الرعاية الصحية فيما يتعلق بتطعيمي |
|  |  |  |  |  | أنا قلق بشأن الآثار الجانبية للتطعيمات |
|  |  |  |  |  | لا احتاج إلى التطعيم للأمراض التي لم تعد منتشرة حالياً |
