# Supplementary material for: Adult Vaccine Hesitancy Scale in Arabic and French: Protocol for Translation and Validation in the World Health Organization Eastern Mediterranean Region
Source: JMIR Res Protoc. 2022 Apr 12;11(4):e36928. doi: 10.2196/36928 (PMC9007230; doi:10.2196/36928)
Supplement: Multimedia Appendix 2 [file resprot_v11i4e36928_app2.docx]

**Appendix II**

**French translation of aVHS**

**Unvalidated version**

| **Questions** | **Pas du tout** | **Peut-être** | **Je ne sais pas** | **Oui** | **Absolument!** |
| --- | --- | --- | --- | --- | --- |
| **Les vaccins sont importants pour ma santé** |  |  |  |  |  |
| **Les vaccins sont efficaces** |  |  |  |  |  |
| **Etre vacciné est important pour la santé des gens autour de soi** |  |  |  |  |  |
| **Tous les vaccins de base recommandés par le gouvernement sont salutaires** |  |  |  |  |  |
| **Les nouveaux vaccins représentent un risque plus élevé que les anciens vaccins** |  |  |  |  |  |
| **Les informations que je reçois du gouvernement à propos des vaccins sont fiables et crédibles** |  |  |  |  |  |
| **Se faire vacciner est un bon moyen de me protéger contre la maladie** |  |  |  |  |  |
| **Normalement, je suis les conseils de mon docteur ou d’un professionnel de la santé à propos de la vaccination** |  |  |  |  |  |
| **Je suis inquiet à propos de la possibilité d’effets secondaires sévères des vaccins** |  |  |  |  |  |
| **Je n’ai pas besoin d’être vacciné contre des maladies qui ne sont plus courantes** |  |  |  |  |  |
