# Supplementary material for: Adult Vaccine Hesitancy Scale in Arabic and French: Protocol for Translation and Validation in the World Health Organization Eastern Mediterranean Region
Source: JMIR Res Protoc. 2022 Apr 12;11(4):e36928. doi: 10.2196/36928 (PMC9007230; doi:10.2196/36928)
Supplement: Multimedia Appendix 3 [file resprot_v11i4e36928_app3.docx]

Original English version of the aVHS.

| **Questions** | **Strongly Disagree** | **Somewhat Disagree** | **Undecided** | **Somewhat Agree** | **Strongly Agree** |
| --- | --- | --- | --- | --- | --- |
| **Vaccines are important for my health** |  |  |  |  |  |
| **Vaccines are effective** |  |  |  |  |  |
| **Being vaccinated is important for the health of others in my community** |  |  |  |  |  |
| **All routine vaccinations recommended by the government are beneficial** |  |  |  |  |  |
| **New vaccines carry more risks than older vaccines** |  |  |  |  |  |
| **The information I receive about vaccines from the government is reliable and trustworthy** |  |  |  |  |  |
| **Getting vaccines is a good way to protect me from disease** |  |  |  |  |  |
| **Generally, I do what my doctor or healthcare provider recommends about vaccines for me** |  |  |  |  |  |
| **I am concerned about serious adverse effects of vaccines** |  |  |  |  |  |
| **I do not need vaccines for diseases that are not common anymore** |  |  |  |  |  |
